# Independent genomewide screens identify the tumor suppressor *VTRNA2-1* as a human epiallele responsive to periconceptional environment

**DOI:** 10.1186/s13059-015-0660-y

**Published:** 2015-06-11

**Authors:** Matt J Silver, Noah J Kessler, Branwen J Hennig, Paula Dominguez-Salas, Eleonora Laritsky, Maria S Baker, Cristian Coarfa, Hector Hernandez-Vargas, Jovita M Castelino, Michael N Routledge, Yun Yun Gong, Zdenko Herceg, Yong Sun Lee, Kwanbok Lee, Sophie E Moore, Anthony J Fulford, Andrew M Prentice, Robert A Waterland

**Affiliations:** MRC International Nutrition Group at London School of Hygiene & Tropical Medicine, Keppel Street, London, WC1E 7HT UK; MRC Keneba, MRC Unit, Atlantic Boulevard, Fajara, P. O. Box 273, Banjul The Gambia; USDA/ARS Children’s Nutrition Research Center, Department of Pediatrics, Baylor College of Medicine, Houston, Texas 77030 USA; Veterinary Epidemiology, Economics and Public Health Group, Royal Veterinary College, Hawkshead Lane, Hatfield, Hertfordshire AL9 7TA UK; International Livestock Research Institute, Old Naivasha Rd, Nairobi, 00100 Kenya; Department of Molecular & Cell Biology, Baylor College of Medicine, Houston, Texas 77030 USA; Epigenetics Group, International Agency for Research on Cancer (IARC), 150 Cours Albert Thomas, 69372 Lyon, CEDEX 08 France; School of Medicine, University of Leeds, Leeds, LS2 9JT UK; Institute for Global Food Security, Queen’s University Belfast, Belfast, BT9 5AG UK; Department of Biochemistry and Molecular Biology, University of Texas Medical Branch, Galveston, Texas 77555 USA; MRC Human Nutrition Research, Elsie Widdowson Laboratory, 120 Fulbourn Road, Cambridge, CB1 9NL UK; Department of Molecular & Human Genetics, Baylor College of Medicine, Houston, Texas 77030 USA

## Abstract

**Background:**

Interindividual epigenetic variation that occurs systemically must be established prior to gastrulation in the very early embryo and, because it is systemic, can be assessed in easily biopsiable tissues. We employ two independent genome-wide approaches to search for such variants.

**Results:**

First, we screen for metastable epialleles by performing genomewide bisulfite sequencing in peripheral blood lymphocyte (PBL) and hair follicle DNA from two Caucasian adults. Second, we conduct a genomewide screen for genomic regions at which PBL DNA methylation is affected by season of conception in rural Gambia. Remarkably, both approaches identify the genomically imprinted *VTRNA2-1* as a top environmentally responsive epiallele. We demonstrate systemic and stochastic interindividual variation in DNA methylation at the *VTRNA2-1* differentially methylated region in healthy Caucasian and Asian adults and show, in rural Gambians, that periconceptional environment affects offspring *VTRNA2-1* epigenotype, which is stable over at least 10 years. This unbiased screen also identifies over 100 additional candidate metastable epialleles, and shows that these are associated with *cis* genomic features including transposable elements.

**Conclusions:**

The non-coding *VTRNA2-1* transcript (also called *nc886*) is a putative tumor suppressor and modulator of innate immunity. Thus, these data indicating environmentally induced loss of imprinting at *VTRNA2-1* constitute a plausible causal pathway linking early embryonic environment, epigenetic alteration, and human disease. More broadly, the list of candidate metastable epialleles provides a resource for future studies of epigenetic variation and human disease.

**Electronic supplementary material:**

The online version of this article (doi:10.1186/s13059-015-0660-y) contains supplementary material, which is available to authorized users.

## Background

Epigenetic mechanisms are established during development and stably regulate gene expression potential in differentiated cells [1]. A fundamental outstanding question is whether and how interindividual epigenetic variation affects risk of disease [2,3]. A major focus is DNA methylation, which in mammals occurs predominantly at cytosines within CpG dinucleotides. Developmental establishment of CpG methylation can be influenced by environment [4,5], and once established, CpG methylation is mitotically heritable and normally highly stable [6]. Elucidating the role of epigenetic variation in human disease is complicated, however, by the fact that epigenetic processes are inherently tissue-specific, and can themselves be altered by disease [7,8]. A potential way to circumvent these complications is to identify epigenetic marks that are established in the very early embryo and maintained during subsequent differentiation, thus affecting all germ layer lineages.

Accordingly, in this study we employed two different approaches to identify DNA methylation changes that are induced by periconceptional environment. First, we performed a genomewide search for metastable epialleles (MEs) in healthy Caucasian adults. MEs are genomic regions at which DNA methylation is established stochastically in the early embryo, leading to systemic (cross-tissue) interindividual variation in epigenetic regulation that is not mediated by genetic variation [9]. Establishment of epigenotype at MEs has previously been shown to be affected by maternal nutrition around the time of conception [10-12]. Second, we used genomewide DNA methylation profiling to study a population in rural Gambia, wherein seasonal variations in food supply and metabolic demand provide a natural experiment by which to study the effect of periconceptional environment (including maternal nutritional status) on epigenetic development in the offspring [13]. These two independent and complementary genomewide screens convergently identified the gene encoding the small non-coding RNA *VTRNA2-1* as the lead candidate environmentally responsive epiallele. *VTRNA2-1* (also called *nc886*) appears to act as a tumor suppressor gene subject to epigenetic silencing by promoter methylation. Elevated methylation at *VTRNA2-1* predicts poor prognosis in leukemia [14], and lung [15] and esophageal cancer [16]. *VTRNA2-1* is genomically imprinted, with preferential methylation on the maternally inherited allele [17,18]. By assaying DNA methylation in peripheral blood mononuclear cells, Treppendahl *et al*. reported that about 25% of healthy individuals exhibit hypomethylation on both alleles of *VTRNA2-1* [14], suggesting polymorphic imprinting. Here we report data indicating that polymorphic imprinting at *VTRNA2-1* is not regulated by *cis* genetic variation, but is affected by maternal environment around the time of conception, occurs systemically, and is highly stable over many years. Our findings provide a plausible causal pathway to explain previous observations that season of birth predicts adult mortality from infection-related causes in rural Gambians [19].

## Results

### Genomewide screen for human metastable epialleles

**Figure 1:**
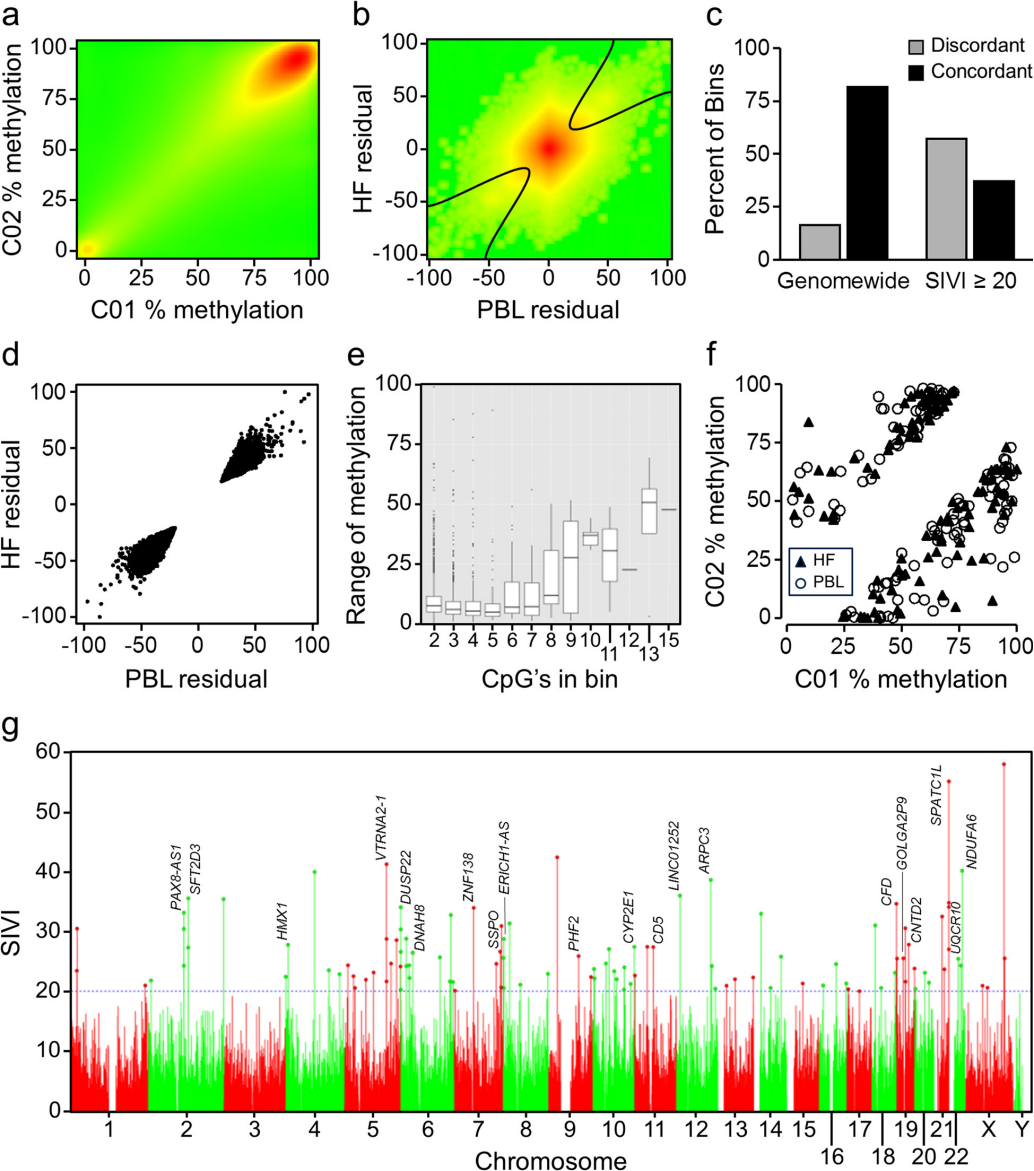
Genomewide screen for human MEs. **(a)** DNA methylation in PBL is highly correlated across the two individuals included in the screen, C01 and C02. The density plot summarizes all 4.1 million 200 bp bins that were covered by sufficient read depth in both samples (R^2^ = 0.926). **(b)** Interindividual DNA methylation residuals (C01-C02) in HF versus those in PBL; 3.9 million 200 bp bins were informative in all four samples. The hyperbola delineates regions containing potential MEs. **(c)** Genomewide, most bins showed no evidence of genetic discordance between the two individuals. Regions of systemic interindividual variation (SIVI ≥20), however, were enriched for interindividual genetic variation. **(d)** HF versus PBL interindividual residual plot for the 4,852 filtered ME bins (SIVI ≥20, no genetic variation, no segmental duplication). The SIVI algorithm effectively targeted the regions indicated in panel (b). **(e)** Targeted analysis of Blueprint Epigenome data (DNA methylation in monocytes of six healthy individuals); ME bins with six or more CpG sites exhibit greatest interindividual variation. **(f)** Interindividual discordance of DNA methylation (C02 versus C01) of the 109 ME bins containing 6 or more CpG sites. **(g)** Manhattan plot of SIVI for all 200 bp bins with 6 or more CpG sites. Bins with SIVI ≥20 (candidate MEs) are crowned; gene-associated bins with SIVI ≥25 are labeled.

As a first approach to identify genomic regions that are epigenetically labile to periconceptional environment, we performed a genomewide screen for human MEs. Improving upon our reduced-representation screen for systemic interindividual variation in DNA methylation [20], we performed genomewide bisulfite sequencing (Bisulfite-seq) on peripheral blood lymphocyte (PBL) and hair follicle (HF) DNA (mesodermal and ectodermal lineages, respectively) from two healthy male US Caucasian adults (C01 and C02) [21]. Our analysis focused on the 6.2 million 200 base pair (bp) genomic bins containing at least 2 CpG sites (hereafter referred to as ‘bins’) [21]. As expected, bin-specific methylation was highly correlated across the two individuals in both PBL (Figure 1a) and HF (Figure S1 in Additional file 1). We formulated a systemic interindividual variation index (SIVI) to identify genomic regions at which interindividual methylation differences are concordant in both tissues (Figure 1b; Table S1 in Additional file 2). Since genetic differences are a major determinant of interindividual epigenetic variation [7], we were not surprised to find that regions of high SIVI (≥20) were enriched for discordant SNPs (*P* < 10^-10^, chi-squared test) (Figure 1c). To focus on putative stochastic effects, subsequent analyses were restricted to the 4,852 high-SIVI bins with no evidence of genetic variation (Figure 1d; Table S2 in Additional file 2). To externally validate interindividual variation in these regions, we performed a targeted analysis of genomewide CpG methylation calls in monocyte DNA from six healthy individuals from the BluePrint Epigenome project [22]. Above a threshold of five CpG sites per bin, the range of bin-specific methylation among these individuals increased and was correlated with CpG density (Figure 1e). We therefore considered only the 109 high-SIVI bins containing ≥6 CpG sites as the most reliable candidate MEs (Figure 1f,g; Table S3 in Additional file 2).

### Genomic features of regions flanking candidate metastable epialleles

**Figure 2:**
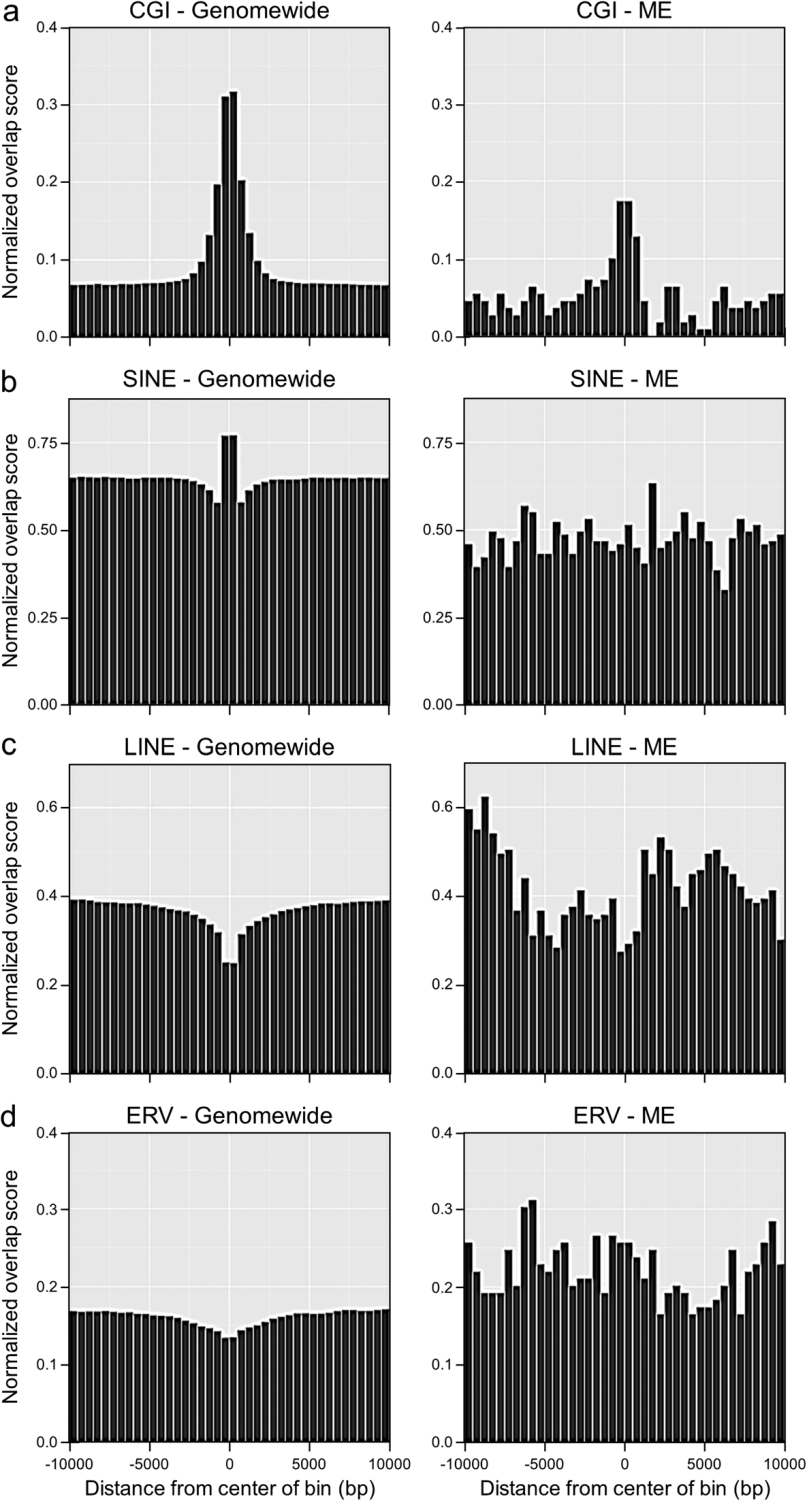
Distribution of CGIs and repetitive elements in ME versus non-ME genomic regions. In each pair of plots, 20 kb regions centered on ME bins (SIVI ≥ 20, n = 109, right) are compared with 20 kb regions centered on comparable non-ME bins genomewide (SIVI = -5 to 5, n = 298,979, left). For each 500 bp window, the normalized overlap score is the number of elements that overlap such windows, divided by the total number of bins. **(a)** ME regions are slightly depleted of CGIs (*P* = 2.5 × 10^-6^). **(b)** ME regions are depleted of SINE elements (*P* = 2.5 × 10^-28^). **(c)** ME regions are enriched for LINE elements (*P* = 7.0 × 10^-8^). **(d)** ME regions are enriched for ERVs (*P* = 3.5 × 10^-15^). All *P*-values based on chi-squared test.

Relative to low-SIVI genomewide bins, the 109 candidate MEs (Figure 1g) were approximately three-fold enriched at subtelomeric regions (*P* = 7.4 × 10^-5^; chi-squared test). Gene ontology analysis indicated that the 64 genes proximal to these bins were not associated with any particular biological process, function, or cellular component. To evaluate associations with sequence features, we compared the 109 candidate ME bins with a genomewide reference set of 298,979 non-ME bins (all with ≥6 CpG sites, SIVI score between -5 and +5, and no evidence of genetic variation), focusing on 20 kb windows centered on each. Genomic regions flanking MEs were depleted of CpG islands (CGIs) and short interspersed nuclear elements (SINEs) (Figure 2a,b), and enriched for long interspersed nuclear elements (LINEs) and endogenous retroviruses (ERVs) (Figure 2c,d). The differences in SINE and ERV content were most dramatic; relative to regions flanking non-ME bins, the 20 kb windows centered on MEs exhibited, on average, a 26.9% depletion of SINEs (*P* = 2.5 × 10^-28^) and a 38.5% enrichment in ERVs (*P* = 3.5 × 10^-15^).

### Validation of epigenetic metastability at *VTRNA2-1*

**Figure 3:**
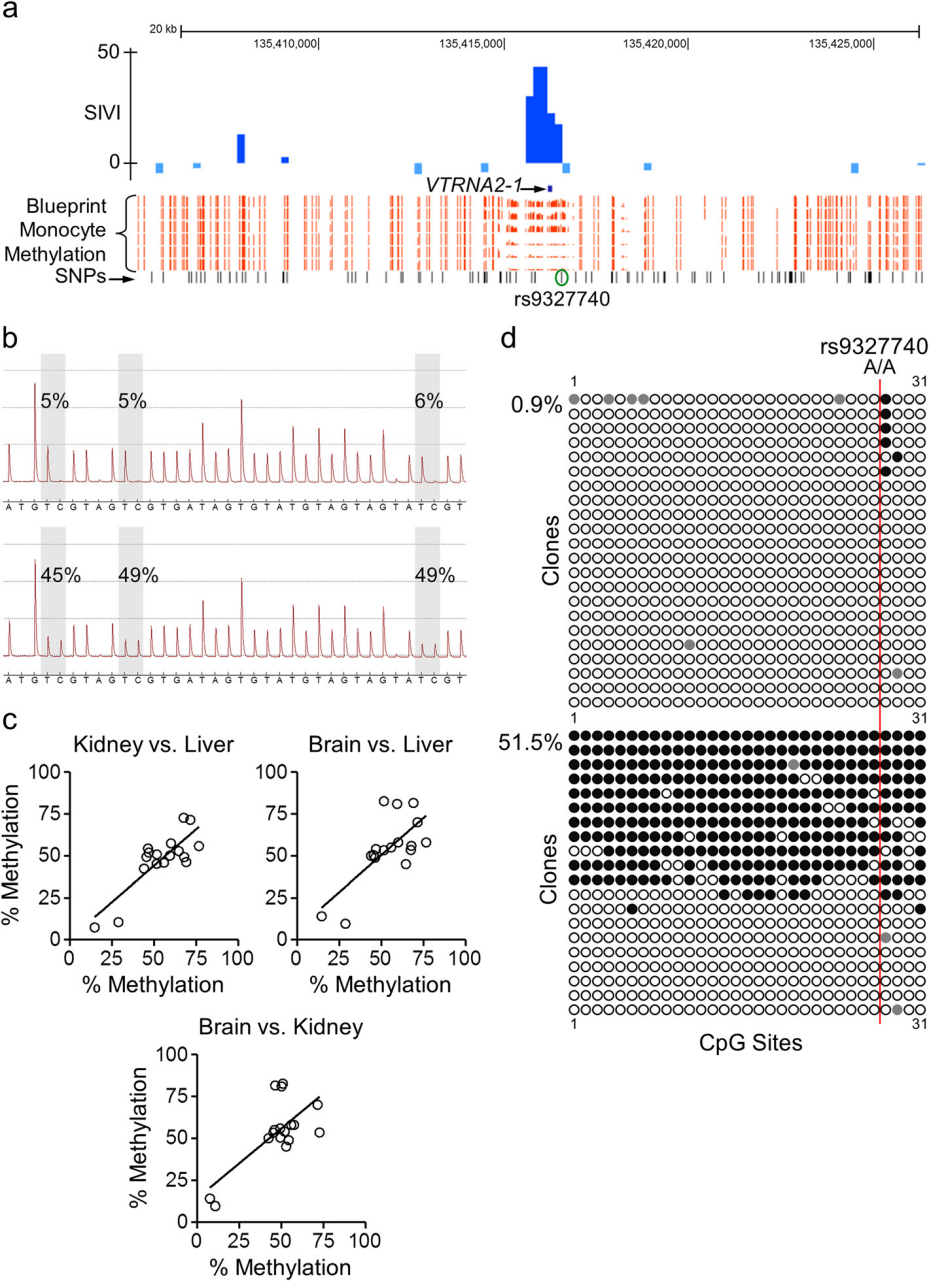
Interindividual epigenetic variation at *VTRNA2-1.* **(a)** UCSC browser shot of the *VTRNA2-1* region on chromosome 5. A cluster of five bins with high positive SIVI (top track) overlaps *VTRNA2-1*. Blueprint Epigenome DNA methylation data on monocytes from healthy individuals (orange) confirm interindividual variation in this same region. **(b)** Bisulfite pyrosequencing results for two individuals with discordant *VTRNA2-1* methylation. T/C polymorphisms resulting from bisulfite conversion at three CpG sites are highlighted in gray. **(c)** Inter-tissue correlations of *VTRNA2-1* methylation across kidney, liver, and brain of 17 Asian cadavers confirm systemic nature of interindividual variation. **(d)** Clonal bisulfite sequencing data on PBL DNA of two Gambian individuals (both A/A at SNP rs9327740) confirm pyrosequencing data and suggest interindividual variation in *VTRNA2-1* methylation is not driven by local genetic variation. Columns and rows correspond to CpG sites and individual clones, respectively. Filled circles indicate methylation; gray circles indicate missing data.

**Figure 4:**
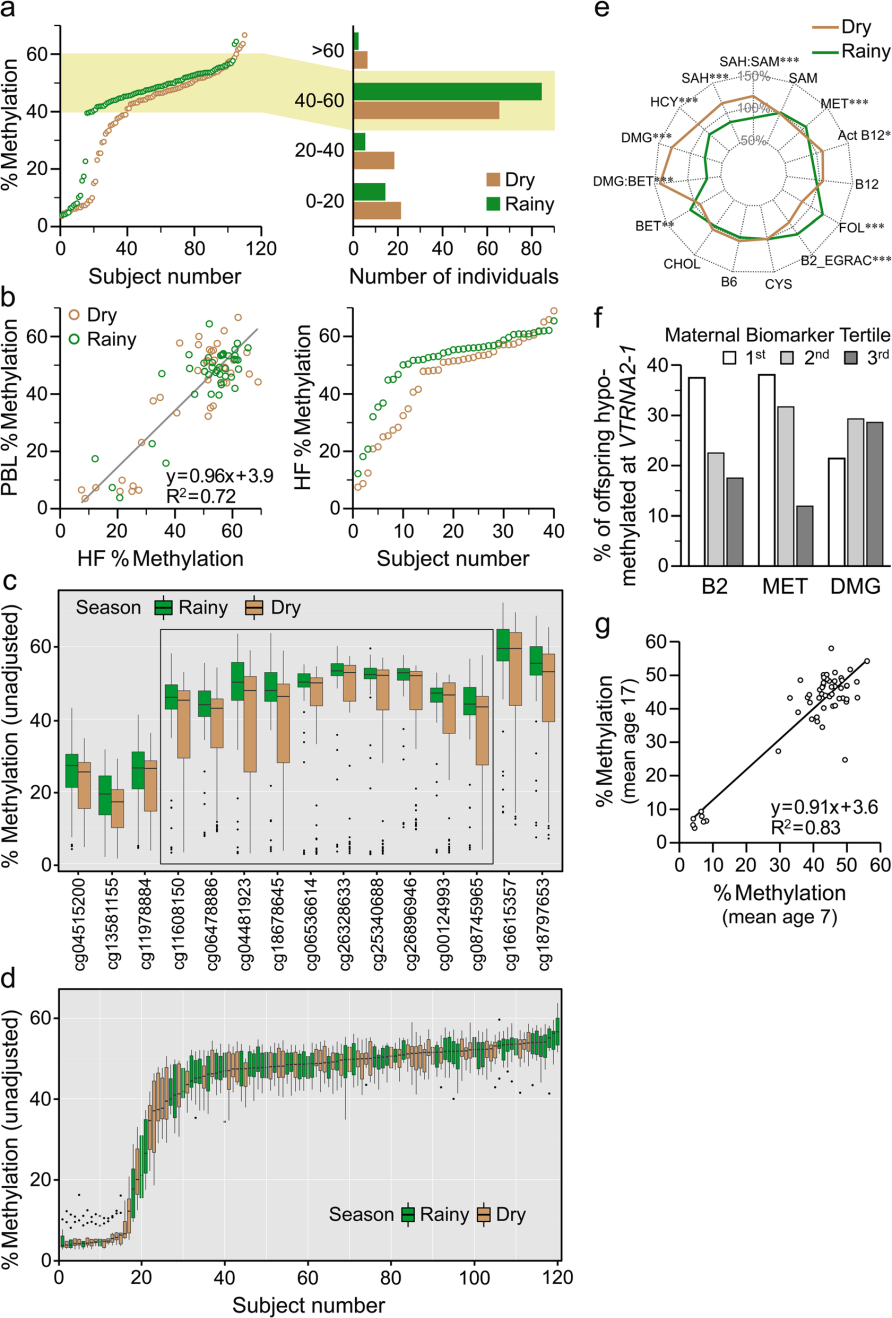
Season of conception (SoC) and maternal periconceptional nutritional status predict methylation at *VTRNA2-1*. **(a)** Bisulfite pyrosequencing data on 215 Gambian children according to SoC. The rank plot (left) highlights the markedly different distribution according to SoC. The histogram (right) shows that individuals conceived in the dry season are under-represented for intermediate methylation expected at an imprinted locus (40 to 60%, highlighted) and over-represented for hypomethylation (*P* = 0.004). **(b)** In 80 Gambian infants with pyrosequencing data on both HF and PBL (left), *VTRNA2-1* methylation in HF is highly correlated with that in PBL. Rank plot of average *VTRNA2-1* methylation in HF of Gambian infants (right) shows that the SoC effect in HF is similar to that in PBL. **(c)** 450k array data on 120 Gambian children, according to SoC. Shown are 15 CpGs mapping to the *VTRNA2-1* locus. The box highlights 10 CpGs corresponding to the imprinted DMR. The SoC effect on hypomethylation spans the entire imprinted DMR (*P* = 0.02, chi-squared test). **(d)** Rank plot of 450k array data at *VTRNA2-1*. Each box represents the methylation values across the 10 CpG sites spanning the imprinted DMR for one individual. **(e)** Seasonal variation in 13 methyl donor-related biomarkers and associated derivatives, back-extrapolated to time of conception and adjusted for gestation age (n = 164 pregnant mothers) [10]. Biomarkers are expressed as percentage of bi-season geometric mean. ANOVA *P*-values of seasonal differences: *<0.05; **<0.01, ***<0.001. **(f)** Maternal nutritional status biomarkers around the time of conception predict *VTRNA2-1* hypomethylation (<40%) in her infant. Low maternal vitamin B2 or methionine (MET) status increases risk of *VTRNA2-1* hypomethylation (*P* = 0.05 and *P* = 0.01, respectively). Low maternal dimethylglycine (DMG) is protective (*P* = 0.05). **(g)** Repeat measurements by bisulfite pyrosequencing in 55 Gambians indicate that *VTRNA2-1* methylation in PBL is highly stable over a period of 10 years.

To identify the strongest candidates at which epigenetic metastability is likely to affect transcription, we prioritized genes associated with multiple proximal MEs. The region flanking the small non-coding transcript *VTRNA2-1* was exceptional in this regard; five adjacent high-SIVI bins encompass the gene (Figure 3a). Interindividual variation in DNA methylation in this region was confirmed by publicly available data from the Blueprint Epigenome project [22] (Figure 3a), and quantitative bisulfite pyrosequencing (Figure 3b) across endodermal (liver), mesodermal (kidney) and ectodermal (brain) tissues of Vietnamese cadavers (Figure 3c) indicated that it occurs systemically (consistent with establishment prior to gastrulation). Clonal bisulfite sequencing data in two individuals matched for genotype at the nearest common SNP (rs9327740) illustrate interindividual variation at *VTRNA2-1* in the absence of local genetic variation (Figure 3d), consistent with its identification as an ME. To test more comprehensively for genetic effects on methylation at *VTRNA2-1* we analyzed data from a recent genomewide study of human methylation quantitative trait loci (mQTL) in 132 lymphoblastoid cell lines representing Northern/Western European (CEU) and West African (YRI) individuals [23]. Remarkably, these cell lines indicated a bimodal distribution of individual methylation values at *VTRNA2-1* (Figure S2 in Additional file 1) very similar to that in primary tissues (Figure 3c). This variation was not significantly associated with any SNPs within 100 kb of the locus (the predominant range for strong mQTL) [23], indicating that interindividual variation in *VTRNA2-1* methylation is not genetically mediated. Further, in PBL samples from Gambian children [10,20], we found evidence that season of conception (SoC) affects establishment of *VTRNA2-1* methylation. Those conceived at the peak of the dry season (n = 110) were significantly more likely to exhibit hypomethylation (<40%) at the *VTRNA2-1* differentially methylated region (DMR; *P* = 0.004) than those conceived in the rainy season (n = 105) (Figure 4a). Similar results were obtained in HF (Figure 4b), indicating that the environmental effect on *VTRNA2-1* epigenotype occurred in the early embryo and was maintained during differentiation of somatic lineages.

### Genomewide screen for DNA methylation changes associated with season of conception in rural Gambians identifies *VTRNA2-1* as the top hit

In subsequent studies we took a completely different approach to identify genomic regions that are epigenetically labile to periconceptional environment, using the Illumina HumanMethylation450 BeadChip array [24] to search genomewide (467,264 CpG sites) for effects of SoC on PBL DNA methylation in 120 Gambian infants [10]. Our pipeline for quality control and pre-processing of the raw methylation data was validated by comparing estimated methylation (beta) values from the 450k array with those obtained from pyrosequencing at five ME loci present on the 450k array (Figure S3 in Additional file 1). Because methylation at neighboring CpG sites is correlated, and biologically relevant variation in methylation often involves clusters of CpG sites [25], we tested for effects across genomic regions rather than at individual probes. We searched for ‘SoC-DMRs’ using the ‘bump hunting’ method [26], with probes adjacent to SNPs common in populations of African ancestry removed, and with sex as an adjustment covariate. Since analysis of leukocyte composition [27] suggested subtle seasonal effects on proportion of CD4^+^ T cells and natural killer cells (Figure S4 in Additional file 1), we also included leukocyte composition as an adjustment covariate. The top 10 SoC-DMRs by permutation *P*-value are presented in Table 1. We were surprised to find that *VTRNA2-1* was the top-ranking SoC-DMR and alone survived multiple testing correction (*P* = 2.0 × 10^-5^; FWER-adjusted *P* = 0.009). Whereas our pyrosequencing data (Figure 4a) focused on only three CpG sites, the 450k results (Figure 4c) showed that the SoC effect on methylation spans the entire *VTRNA2-1* imprinted DMR (represented by the 10 CpG sites with methylation close to 50%). Despite this interindividual variation, methylation across the entire *VTRNA2-1* DMR was tightly regulated within each individual (Figure 4d). The observed distribution is suggestive of a failure to maintain methylation at the normally silenced allele in some children, especially those conceived in the dry season, although confirmation of this would require further studies. Notably, the last 10 CpG sites in the *VTRNA2-1* SoC DMR (Figure 4c, cg04481923 to cg18797653) correspond to those recently reported to show differences in tumor versus normal tissues, using the same array platform [18].

**Table 1:** **The 10 most significant SoC-associated DMRs (SoC-DMRs) identified by the bump hunting analysis**

**Chr**	**SoC-DMR start**	**SoC-DMR end**	**Mean coefficient**	**Probes in SoC-DMR**	**Probes in cluster**	**SoC** ***P*** **-value**	**SoC** ***P*** **-value (FWER)**	**Gene**	**Annotation**
5	135,415,762	135,416,613	0.61	15	15	2.0E-05	0.009	*VTRNA2-1*	Overlaps TSS
2	113,992,762	113,993,313	0.47	8	8	5.5E-04	0.228	PAX8*	Intron/exon
5	23,507,030	23,507,752	0.36	12	13	8.7E-04	0.349	*PRDM9*	Overlaps TSS
6	32,729,442	32,729,847	0.14	20	36	1.6E-03	0.576	*HLA-DQB2*	Intron/exon
17	17,109,570	17,110,120	0.38	8	11	1.8E-03	0.578	*PLD6*	Overlaps TSS
6	29,648,345	29,649,024	0.27	14	18	1.8E-03	0.601	ZFP57*	~3 kb upstream of TSS
6	151,646,312	151,647,133	0.30	9	9	2.8E-03	0.733	*AKAP12*	Overlaps TSS
12	57,040,045	57,040,204	0.36	4	9	3.2E-03	0.782	ATP5B*	Promoter
5	191,242	192,103	0.26	10	11	3.8E-03	0.848	*LRRC14B*	Overlaps TSS
13	36,944,640	36,944,649	0.36	2	8	4.4E-03	0.864	*SPG20*	Promoter

Analysis includes adjustment for sex and estimated white blood cell composition. Gene annotations are those provided by Illumina, except for entries marked with an asterisk, for which overlapping or proximal genes are listed. TSS, transcription start site.

All of the top 10 SoC-DMRs (Table 1) showed lower methylation in the dry relative to the rainy season (that is, a positive coefficient), consistent with our previous studies focused on candidate MEs [10,20]. Plots of methylation by SoC for the second and third highest ranking SoC-DMRs (*PAX8* and *PRDM9*) are provided as examples (Figure S5a,b in Additional file 1). Importantly, *VTRNA2-1* was not the only candidate ME that showed SoC effects in the 450k data. *PAX8* and *AKAP12*, identified as candidate MEs in our previous reduced-representation screen [20], were among the top 10 SoC-DMRs (Table 1), reinforcing the notion that developmental establishment of DNA methylation at MEs is particularly sensitive to periconceptional environment.

### Maternal periconceptional nutrition predicts offspring hypomethylation at *VTRNA2-1*, which is stable for many years

Seasonal differences in maternal nutritional status affecting one-carbon metabolism (Figure 4e) suggest a potential mechanism to explain the SoC effect on *VTRNA2-1* methylation. To test this, each of 13 maternal nutritional status biomarkers sampled in early pregnancy [10] was evaluated as a potential predictor of *VTRNA2-1* hypomethylation in her infant (<40% by pyrosequencing) [28] (Table S4 in Additional file 2). Low maternal vitamin B2 (riboflavin) and methionine (MET) specifically around the time of conception predicted *VTRNA2-1* hypomethylation in her infant, whereas low maternal plasma dimethylglycine (DMG) protected against hypomethylation (Figure 4f; *P* = 0.05, 0.01, and 0.05 respectively). Each of these associations appears to follow a dose-response relationship, but larger datasets will be needed to confirm them and model the relevant methyl donor pathways. The riboflavin finding, consistent with our previous observations across a panel of MEs [10], is particularly noteworthy; riboflavin is required for synthesis of flavin-adenine dinucleotide, an essential cofactor for methylenetetrahydrofolate reductase (MTHFR), a rate-limiting enzyme in one-carbon metabolism. Riboflavin deficiency is very common in low-income populations with low intakes of dairy and animal foods, including rural Gambia.

In order for a nutritionally mediated epigenetic change in early life to affect risk of disease in adulthood, such marks must persist for many years. To test the temporal stability of *VTRNA2-1* methylation, we obtained serial PBL DNA samples from 55 rural Gambians, spanning a 10-year period. Average *VTRNA2-1* methylation was generally stable from approximately 7 to 17 years of age (Figure 4g), indicating that individual patterns of methylation at the locus, once established in the early embryo, persist to adulthood.

## Discussion

Previous studies have cataloged interindividual variation in DNA methylation in specific cell types [29,30]. Our approach, screening for concordant variation in multiple tissues representing different embryonic lineages, is unique in enabling the identification of systemic interindividual variation [20]. Here, using Bisulfite-seq to analyze the PBL and HF DNA methylomes of two Caucasian adults, we performed the first truly genomewide screen for human MEs. We followed this with a genomewide search for SoC effects on DNA methylation in Gambian infants. *VTRNA2-1* was a top hit by both approaches. The convergence of these two independent genomewide screens at this one locus positions *VTRNA2-1* as a potential major indicator of early environmental effects on epigenetic regulation in humans. Interindividual variation in *VTRNA2-1* methylation has been reported in peripheral blood mononuclear cells of healthy individuals [14] and in adjacent normal tissues collected during tumor biopsies [18]. Neither of those previous studies, however, demonstrated concordant interindividual variation across multiple tissues from the same individuals. (Although Romanelli *et al*. [18] showed four examples of allelic methylation in both placenta and cord blood of the same individuals, all were approximately 50% methylated - that is, no interindividual variation.) Here, by studying liver, kidney, and brain of Vietnamese cadavers, and PBL and HF of healthy Caucasians and Gambians we showed that hypomethylation, suggestive of loss of imprinting at *VTRNA2-1*, occurs systemically in specific individuals in diverse populations. Further, we demonstrated that this is partially determined by periconceptional environment, and is stable over at least 10 years.

The unbiased nature of our ME screen enabled us to characterize genomic features associated with epigenetic metastability. Rather than localized differences, our analysis identified substantial depletion of CGIs and SINEs, and enrichment of LINEs and ERVs across genomic regions at least 10 kb upstream and downstream of candidate MEs (Figure 2). Since transposable elements are important determinants of regional DNA methylation patterns [31], these widespread sequence features likely contribute to the stochastic epigenetic variation at these loci. The enrichment of ERVs is particularly noteworthy in that all documented mouse MEs are associated with retrotransposition of an intracisternal A particle (a murine ERV) [9].

Although genomically unbiased, our ME screen does have limitations. Due to the expense of performing Bisulfite-seq, we profiled two tissues from only two individuals. More MEs are likely to be discovered by future screens including more individuals; including at least three tissues (representing all three germ layer lineages) may also be advantageous. As expected, several of the genes identified as candidate MEs in our previous reduced-representation screen [20] were also identified here. Our results also corroborate those of another ME screen based on our multiple-tissue approach. Using the Illumina 450k array to profile DNA methylation in peripheral blood leukocytes and colonic mucosa of 10 children, Harris *et al*. [32] identified 1,776 CpG sites associated with 1,013 genes as candidate MEs. Of the 1,013 genes they identified, 198 (19.5%) overlap with those associated with the unfiltered candidate MEs we identified (Table S1 in Additional file 2), many more than expected by chance (*P* = 2.1 × 10^-8^; chi-squared test). Moreover, of the 1,776 CpGs Harris *et al*. identified as candidate MEs, four are within or near our top 10 SoC DMRs (Table S7 in Additional file 2). Among these, Harris *et al*. identified one probe (cg04515200) within the *VTRNA2-1* SoC DMR (Figure 4c). Given the low intraindividual variance at *VTRNA2-1* (Figure 4d), we were surprised that a lone probe would be identified as an ME (rather than the entire region). Indeed, examination of the Harris *et al*. data confirmed systemic interindividual variation in methylation across the entire *VTRNA2-1* imprinted DMR (Figure S6 in Additional file 1), suggesting that perhaps the filtering criteria they used were overly conservative. Evidence of a SoC effect at the other three loci (Table S7 in Additional file 2) strengthens their candidacy as MEs.

Of these, the SoC DMR at *ZFP57* is particularly interesting because *ZFP57* plays a key role in maintaining allelic methylation during pre-implantation development [33]. This genomic region (approximately 3 kb upstream of the gene) was identified as a candidate ME by both Harris *et al*. [32] and our current screen (prior to SNP filtering) (Table S1 in Additional file 2). Although it is intriguing that *ZFP57* is among the top SoC DMRs (Table 1), the effect is very subtle (Figure S5c in Additional file 1) and should be interpreted with caution. Nonetheless, in a recent genomewide (450k) study of immune cells from cord blood of newborn infants [34], methylation in this same region was found to be strongly predicted by maternal folate status in late pregnancy. Future studies will be required to determine if this master regulator of allelic methylation is indeed an ME.

This and preceding studies [20,32] have thus far screened for MEs only in Caucasians. We did previously show that candidate MEs identified by our multiple-tissue screen in Caucasians exhibit similar patterns of interindividual DNA methylation variation across Asians and West Africans [20], suggesting that epigenetic metastability is an ancestral feature of the human genome. This conclusion is reinforced here by our data on *VTRNA2-1*. Indeed, our discovery in Gambians of a SoC effect at the *VTRNA2-1* ME (which was identified in Caucasians) is a great strength of this study. Nonetheless, it will be important for future studies to perform the multiple-tissue ME screen in non-Caucasians.

One potential criticism of our approach for identifying SoC DMRs is that our 450k analyses were performed using unfractionated leukocytes. Using the method of Jaffe and Irizarry [27], however, we found evidence of only minor SoC effects on leukocyte composition (Figure S4 in Additional file 1), and our analyses of the 450k data included adjustment for this. An analysis of previous Illumina 450k data on flow-sorted blood cells [27] found no cell type-specific differences in DNA methylation at any of the 15 CpGs comprising the *VTRNA2-1* SoC DMR (Table S5 in Additional file 2). Hence, the SoC effect at *VTRNA2-1* should be unaffected by changes in blood cell composition. Indeed, we found a similar SoC effect in HF and PBL (Figure 4a,b), tissues derived from different germ layer lineages.

In an attempt to focus on interindividual epigenetic variation that is not genetically mediated, we filtered out candidate MEs associated with genetic variation within 200 bp bins. However, genetic variants that influence DNA methylation (mQTL) can operate over vast genomic distances. To evaluate potential longer-range genetic effects on DNA methylation at *VTRNA2-1*, we analyzed genome-wide mQTL data recently reported by Zhang *et al*. [23] on lymphoblastoid cell lines from 132 individuals of European and African origin. The distribution of polymorphic imprinting in these cell lines was impressively similar to that in primary tissues; the 10 CpG sites comprising the *VTRNA2-1* imprinted DMR were hypomethylated in approximately 20% of individuals (Figure S2 in Additional file 1). However, at none of these sites did the study by Zhang *et al*. (currently among the largest genome-wide mQTL screens) detect mQTL. Given the strong bimodal distribution of individual *VTRNA2-1* methylation, if hypomethylation at the locus was regulated by *cis* genetic variation, it should have been detected. Hence, these data argue strongly against interindividual variation at *VTRNA2-1* being genetically mediated. The environmentally mediated effect of SoC on hypomethylation in Gambian children further supports this interpretation.

## Conclusions

Together, our data suggest that the effect of maternal nutrition on DNA methylation at *VTRNA2-1* exhibits all the hallmarks of ‘metabolic imprinting’ [35]: a critical window of sensitivity (in the pre-implantation embryo), a dose-response relation between exposure and outcome, and a persistent effect. Because *VTRNA2-1* is transcriptionally regulated by methylation at its promoter [14,17] and appears to act as a tumor suppressor in various types of cancer [14-16], metabolic imprinting of *VTRNA2-1* DNA methylation is a likely determinant of cancer risk. Moreover, since the *VTRNA2-1* transcript affects PKR-mediated regulation of immune function [36], this early environmental effect on DNA methylation could have far-reaching effects on immune function and might offer an explanation for how season of birth (which maps onto SoC) affects adult mortality from infectious disease in rural Gambians [19,37]. More generally, we anticipate that the list of candidate metastable epialleles we identified will provide a resource for future studies of epigenetic variation and human disease.

## Materials and methods

### Human subjects

Informed written consent was obtained from all subjects prior to participation, and experimental methods complied with the Helsinki declaration. Scientific approval for the Caucasian studies was obtained under IRB protocol H-18849 at the Baylor College of Medicine. For the Gambian studies, the Scientific Coordinating Committee of MRC Unit, The Gambia, granted scientific approval and the joint Gambian Government/MRC Ethics Committee (SCC/EC 1151) and the London School of Hygiene and Tropical Medicine Ethics Committee (EC 5525) granted ethical permission for this study. Sample collection, study populations, and DNA isolation have been previously described [10,20,21].

### Bisulfite-seq, SNP calling from Bisulfite-seq data, and filtering bins based on SNP score

Bisulfite-seq library preparation and sequencing were performed in the Baylor College of Medicine Human Genome Sequencing Center, and read mapping and data processing were performed as previously described [21]. The accuracy of our methylation calls by Bisulfite-seq has been quantitatively validated [21]. Combined bisulfite-seq data from HF and PBL samples from two individuals (C01 and C02) [21] were used to determine SNP scores for 200 bp bins genomewide. Reads were mapped to hg19 with BISMARK [38] and BisSNP (v.0.82.2) [39] was run to call SNPs on each combined sample (PBL + HF) using the default settings and dbSNP 135. The VCFpostprocess tool of BisSNP was used with the default settings to filter the raw SNP calls. SNP scores were assigned at each locus for which there was a SNP called in either C01 or C02. A score of 1 was assigned to those loci at which both C01 and C02 had the same SNP call. A score of 0 was assigned otherwise. Average SNP scores were calculated for all 200-bp bins genomewide. Genomewide bins were defined as those containing at least 2 CpG sites and which had mapping coverage [21] in at least one of the four C01/C02 HF/PBL samples (N = 5,257,320). A bin’s SNP score was set to 1 if no SNP was called in either individual.

### Formulation of the systemic interindividual variation index

$$ x = \mathrm{P}\mathrm{B}\mathrm{L}\ \mathrm{residual}\ \left(\%\mathrm{meth}\ \mathrm{in}\ \mathrm{C}01\ \hbox{-}\ \%\mathrm{meth}\ \mathrm{in}\ \mathrm{C}02\right) $$

$$ y = \mathrm{H}\mathrm{F}\ \mathrm{residual}\ \left(\%\mathrm{meth}\ \mathrm{in}\ \mathrm{C}01\ \hbox{-}\ \%\mathrm{meth}\ \mathrm{in}\ \mathrm{C}02\right) $$

$$ \mathrm{SIVI}=\mathrm{A}+\mathrm{B}+\mathrm{C} $$

Where

$$ \mathrm{A}=\sqrt{\left(\left|x\cdot y\right|\right)}\left(\mathrm{rewards}\ \mathrm{maximal}\ \mathrm{interindividual}\ \mathrm{differences}\right) $$

$$ \mathrm{B}=-sd\left(x,y\right)\left(\mathrm{rewards}\ \mathrm{in}\mathrm{terindividual}\ \mathrm{differences}\ \mathrm{that}\ \mathrm{are}\ \mathrm{similar}\ \mathrm{in}\ \mathrm{both}\ \mathrm{tissues}\right) $$

$$ \mathrm{C}=- \max \left(sd{\left(\% met{h}_{PBL},\% met{h}_{HF}\right)}_{C01},sd{\left(\% met{h}_{PBL},\% met{h}_{HF}\right)}_{C02}\right)\left(\mathrm{rewards}\ \mathrm{consistent}\ \mathrm{percent}\ \mathrm{methylation}\ \mathrm{across}\ \mathrm{both}\ \mathrm{tissues}\right) $$

### Targeted analysis of BluePrint epigenome data

Bisulfite-seq methylation calls from each of six monocyte samples (C000S5, C0010K, C001UY, C004SQ, C005PS, S000RD) from the Blueprint Epigenome project [22] were placed into 200 bp bins and an average methylation score was determined for each bin. Each bin which had a methylation score in at least two samples was then assigned a range score, defined as the difference between the highest and lowest methylation score of all informative samples.

### Comparison of genomic features among metastable epiallele and non-metastable epiallele genomewide bins

The distances from each non-ME and ME bin genomewide were determined for all SINE, LINE, and ERV elements (UCSC RepeatMasker track, hg19) and CGIs (UCSC CpG islands track, hg19). Non-ME bins (N = 298,979) were defined as those having a SIVI score between -5 and 5, a SNP score of 1, no overlap with segmental duplication (UCSC, hg19), and at least six CpG sites. ME bins (N = 109) had a minimum SIVI of 20 but otherwise the same characteristics. A 20 kb window centered on the midpoint of each bin was divided into 40 intervals of 500 bp each. The overlap score is the number of each type of individual elements that overlap the interval. The 'normalized overlap score' was calculated by dividing the raw overlap score by the total number of bins in each set. Chi-squared tests were performed on the raw overlap scores for CGIs and SINE, LINE, and ERV elements. A chi-squared test was also performed to determine the significance of the localization of ME bins to subtelomeric regions (defined as the 1 Mb flanking each 10 kb telomere).

### Bisulfite pyrosequencing

Quantitative analysis of CpG site-specific DNA methylation at *VTRNA2-1* was performed by bisulfite pyrosequencing [40]. The pyrosequencing assay was validated using standards composed of known mixtures of methylated and unmethylated human genomic DNA [41] (Figure S7 in Additional file 1). The primers used were as follows: forward TGAAGGTGTGATAGAAAGTATG, reverse (Biotin) ACATTTTTTTATCCCCATA, sequencing AGTATGGAGGTTGGTTATT.

### 450k array hybridization, data processing, and analysis of the Gambian sample cohort

Sample preparation and hybridization to the Illumina HumanMethylation450 BeadChip arrays were performed at the Genetics Services Platform of the International Agency for Research on Cancer, according to manufacturer’s instructions. Data processing and analysis were performed as follows: 1) pre-filtering and quality control (QC); 2) color adjustment, probe-type bias correction and inter-sample quantile normalization; 3) batch effect correction and evaluation of alternative QC pipelines; 4) data-driven estimation of white blood cell counts; 5) replicate removal and outlier detection; 6) removal of probes close to SNPs; 7) bump hunting analysis.

#### Pre-filtering and quality control

Raw data for 485,577 CpG probes on the 450k array were loaded from IDAT files (n = 124 samples including three technical replicates). Array-wide two-dimensional multi-dimensional scaling plots clustered into two groups representing infant sex, confirming that recorded sex was correct for all samples. We then removed 11,656 probes on X and Y chromosomes, together with 57 SNP probes provided to detect potential sample mix-ups; 473,864 probes remained.

A further probe filtering step was implemented based on probe detection *P*-values. Using a detection *P*-value threshold of *P* = 0.01, the maximum sample failure rate was 0.00383 (sample ID = 9007225117_R06C01; Figure S8 in Additional file 1). This sample also appears as an outlier on principal component analysis plots of array-wide methylation (Figure S9 in Additional file 1) and was removed from all subsequent analysis; n = 123 samples remained. We also removed 6,600 probes that failed in one or more samples (using the same detection *P*-value threshold), leaving 467,264 probes for subsequent analysis. Additional quality control checks (overall signal intensity (M + U) across samples, M-value distributions and multi-dimensional scaling plot excluding X and Y probes) revealed no further issues.

#### Color adjustment, probe-type bias correction and inter-sample quantile normalization

Standard pre-processing adjustments as described in [42] were applied as follows: a) correction for probe color bias (lumi col adj), b) inter-sample quantile normalization (lumi QN), and c) probe type correction using beta mixture quantile dilation (BMIQ) [43]

#### Batch effect correction and evaluation of alternative quality control pipelines

The following alternative QC pipelines with and without correction for batch covariates (sample plate, sample slide, sample position on slide) were considered:

- p1A: col adj + QN + BMIQ + batch correction (using ComBat [44])*
- p1B: col adj + QN + BMIQ + batch correction, adjusting for SoC (using ComBat [44])*
- p2: col adj + BMIQ + adjust for batch covariates on locus-by-locus basis**
- p3: col adj + QN + BMIQ adjust for batch covariates on locus-by-locus basis**
- p4: col adj + QN + BMIQ (no adjustment for batch covariates)
- p5: col adj + BMIQ (no adjustment for batch covariates)

*Batch correction using ComBat can be applied with or without adjusting for the variable of interest (in our case SoC).

**For p2 and p3, batch effects are first estimated in a linear multiple regression including batch covariates and SoC. Adjusted data are then the residual variation after adjusting for estimated batch effects only.

The performance of each pipeline was evaluated by comparing 450k beta values with percentage methylation estimates obtained from pyrosequencing data at five ME loci overlapping 450k CpG probes (Figure S3 in Additional file 1). Each pipeline was further evaluated by comparing 450k beta values between three pairs of technical replicates on the 450k array. Bland-Altman plots [45] illustrating replication performance across the full range of percentage methylation for each of three technical replicates using ‘optimal’ QC pipeline (p4) are presented in Figure S10 in Additional file 1.

#### Summary of QC pipeline evaluation

- The p4 (col adj + QN + BMIQ) and p5 (col adj + BMIQ) approaches gave the best performance when comparing 450k and pyrosequencing methylation estimates at overlapping loci.
- Neither of these methods (p4 or p5) feature direct batch adjustment, although p4 does remove a substantial amount of batch variation through inter-sample QN.
- Regression-based locus-by-locus adjustment for batch covariates gives relatively poor 450k versus pyrosequencing correlations.
- We selected p4 (col adj + QN + BMIQ) as our optimal pipeline. This achieves a Pearson correlation of 0.92 (Spearman R = 0.94) between 450k and pyrosequencing assays, with 88% of beta values differing by less than 15% between the two platforms. These figures compare favorably with a similar analysis [45] performed across 340 CpGs from 4 human breast cancer cell lines (Spearman R = 0.88; 81% with beta difference <15%).

#### Data-driven estimation of white blood cell counts

Interpretation of analyses investigating DMRs or differentially methylated positions (DMPs) in whole blood should be treated with caution, due to the possibility of confounding by white blood cell (WBC) type. We obtained methylation data-driven estimates of WBC-type composition using the method described by Jaffe and Irizarry [27]. These estimates are stable across technical replicates (Figure S11 in Additional file 1).

There is some evidence for potential confounding by WBC in our data. First, WBC type may be weakly associated with SoC (Figure S4 in Additional file 1). Secondly WBC composition is strongly associated with principal components explaining a large portion of genomewide variation in methylation (Table S6 in Additional file 2). We therefore performed our DMR analysis using bump hunting with adjustment for estimated WBC composition. An independent study [27] identified probes on the 450k array that are differentially methylated according to WBC type. None of the probes mapping to *VTRNA2-1* in our study fall within this set (Table S5 in Additional file 2).

#### Replicate removal and outlier detection

For each of the three technical replicates, we remove the replicate with the highest probe fail rate. One outlier is also removed (see ‘Pre-filtering and quality control’ section above), leaving n = 120 samples for final analysis.

#### Removal of probes close to SNPs

450k probes within 10 bp of a common African SNP, defined as ‘AFR’ designated polymorphisms with a minor allele frequency >1% using data from the 1000 Genomes Project [46] were removed. We identified 42,435 such probes, so that 424,829 CpGs remain for the bump hunting analysis. SNP filtering was performed using the R ‘Illumina450ProbeVariants.db’ package.

#### Bump hunting analysis

The bump hunting method [26] measures differential methylation across pre-defined clusters of neighboring CpGs. We used the recommended proximity-based criteria for defining clusters so that each cluster contains a minimum of seven probes on the 450k array, with each probe located within 300 bp of its nearest neighbour. After filtering of SNP-proximal probes (see previous section), this resulted in a total of 10,394 clusters, with a maximum cluster size of 104 CpGs. We performed the bump hunting analysis with methylation pre-adjusted for sex and WBC composition, since the inclusion of adjustment covariates in the bumphunter linear model is not recommended (see [47]). M-values (logit-transformed beta-values) are used in place of untransformed beta-values throughout [48].

The bump hunting method first fits a linear model at each CpG within a cluster, with M-value as the outcome variable and SoC as the predictor variable. A smooth curve (loess) is then fitted to the estimated SoC coefficients across each cluster. Regions where the smoothed estimated coefficients deviate far from zero were considered candidate DMRs. Specifically, these are defined as groups of neighboring probes whose smoothed absolute coefficients exceed the 99th percentile of all estimated coefficients. To form a null distribution that accounts for (a) correlations between probes, (b) differences in cluster size and (c) potential non-normal distribution of model errors, this process is repeated 1,000 times with the outcome variables (M-values) permuted. The permutation *P*-value for a specific DMR is then the proportion of candidate DMRs across all permutations that had both a larger mean absolute coefficient and a larger length (number of probes) than the empirically observed DMR. We additionally report FWER-adjusted *P*-values, which are the proportion of permutations having at least one region as extreme as the empirically observed DMR.

### Analysis of associations between infant methylation at *VTRNA2-1* and season of conception and maternal 1-carbon biomarker concentrations

*VTRNA2-1* methylation assayed at three CpG sites by bisulfite pyrosequencing was observed to be highly correlated (mean Spearman R = 0.93). Because percentage methylation values followed a distinct bimodal pattern, we used mean methylation, dichotomized at 40% [28], as our outcome variable. Association of SoC with dichotomized mean methylation was assessed using a Pearson chi-squared test. For testing associations of maternal biomarkers with dichotomized mean methylation, all biomarkers were back-extrapolated to time of conception, adjusted for gestational age, and analyzed in the logarithm, as described previously [10]. Biomarker associations were analyzed in a logistic regression model using the glm function in R.

### Data availability

The Gene Expression Omnibus (GEO) accession number for the raw sequence reads for the four Bisulfite-seq libraries is GSE44806. The GEO accession number for the original 450k data sets is GSE59592.

## Additional files

Additional file 1:
**Supplementary Figures S1 to S11.**
Additional file 2:
**Supplementary Tables S1 to S7.**

## Abbreviations

BMIQ: beta mixture quantile dilation
bp: base pair
CGI: CpG island
DMR: differentially methylated region
ERV: endogenous retrovirus
HF: hair follicle
LINE: long interspersed nuclear element
ME: metastable epiallele
mQTL: methylation quantitative trait locus
PBL: peripheral blood lymphocyte
QC: quality control
QN: quantile normalization
SINE: short interspersed nuclear element
SIVI: systemic interindividual variation index
SoC: season of conception
SNP: single-nucleotide polymorphism
WBC: white blood cell
