# Supplementary material for: Independent genomewide screens identify the tumor suppressor VTRNA2-1 as a human epiallele responsive to periconceptional environment
Source: Genome Biol. 2015 Jun 11;16(1):118. doi: 10.1186/s13059-015-0660-y (PMC4464629; doi:10.1186/s13059-015-0660-y)
Supplement: Additional file 1: — Supplementary Figures S1 to S11. [file 13059_2015_660_MOESM1_ESM.pdf]

Supplementary Information for:

Independent genomewide screens identify the tumor suppressor *VTRNA2-1* as a human epiallele responsive to periconceptional environment

Matt J. Silver[1], Noah J. Kessler[1], Branwen J. Hennig, Paula Dominguez-Salas, Eleonora Laritsky, Maria S. Baker, Cristian Coarfa, Hector Hernandez-Vargas, Jovita M. Castelino, Michael N. Routledge, Yun Yun Gong, Zdenko Herceg, Yong Sun Lee, Kwanbok Lee, Sophie E. Moore, Anthony J. Fulford, Andrew M. Prentice[2], Robert A. Waterland[2]

[1]These authors contributed equally.
[2]Correspondence to: waterland@bcm.edu or Andrew.Prentice@lshtm.ac.uk

This PDF file includes:

Figures S1 to S11

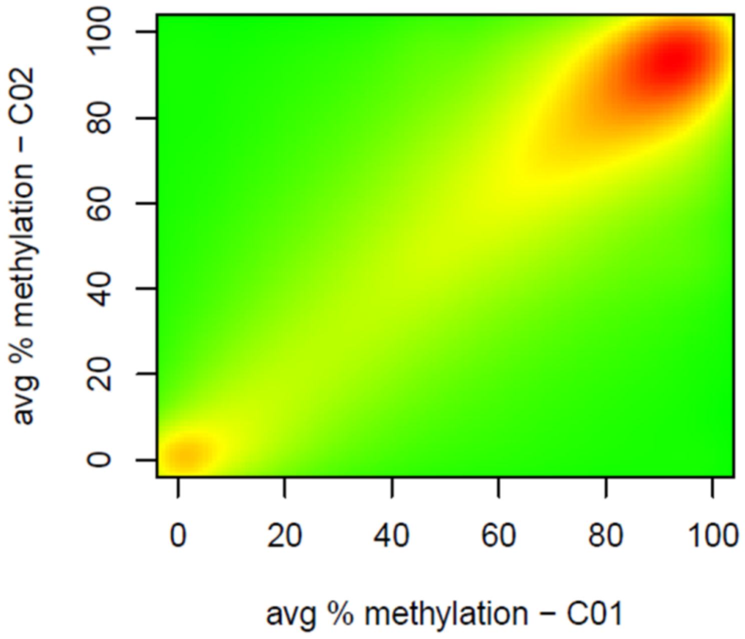

Supplementary Figure 1: Density plot of average methylation (by 200 bp bin) in hair follicle (HF) in individual 2 (C02) vs. individual 1 (C01), for all 4.5M 200 bp bins that were informative in both samples. Genomewide, DNA methylation in HF is highly correlated across the two individuals (R^2=0.930).

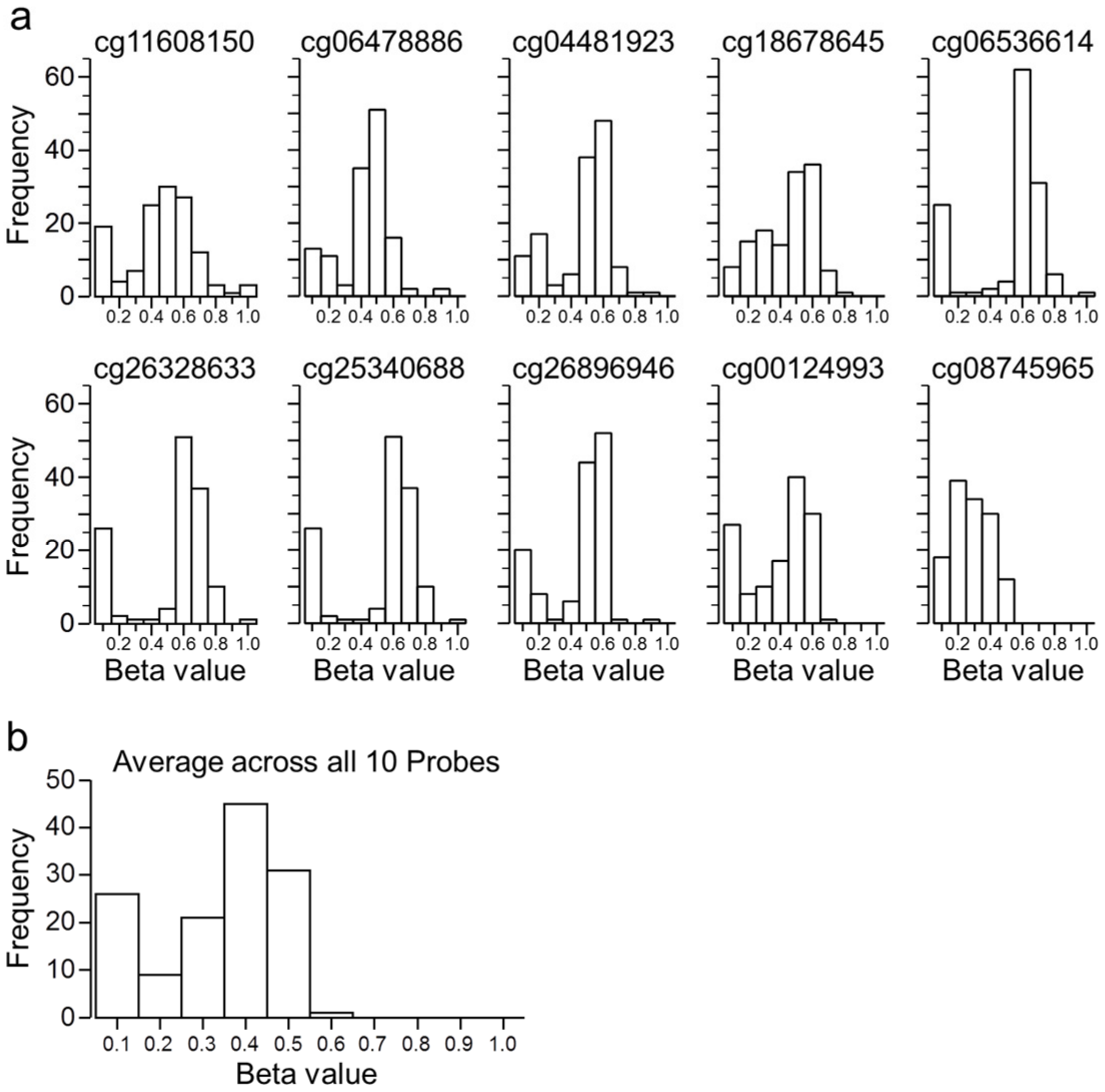

Supplementary Figure 2: Lymphoblastoid cell lines from 132 HapMap individuals display interindividual variation at *VTRNA2-1* similar to what is observed in primary tissues. (Analysis of Illumina 450k data of Zhang et al (*Hum Mol Genet* 2014.) (**a**) At each of the 10 probes corresponding to the *VTRNA2-1* DMR, a wide range of individual methylation values is observed. (**b**) Distribution of individual average methylation across all 10 probes confirms regional interindividual variation, with a strong bimodal distribution.

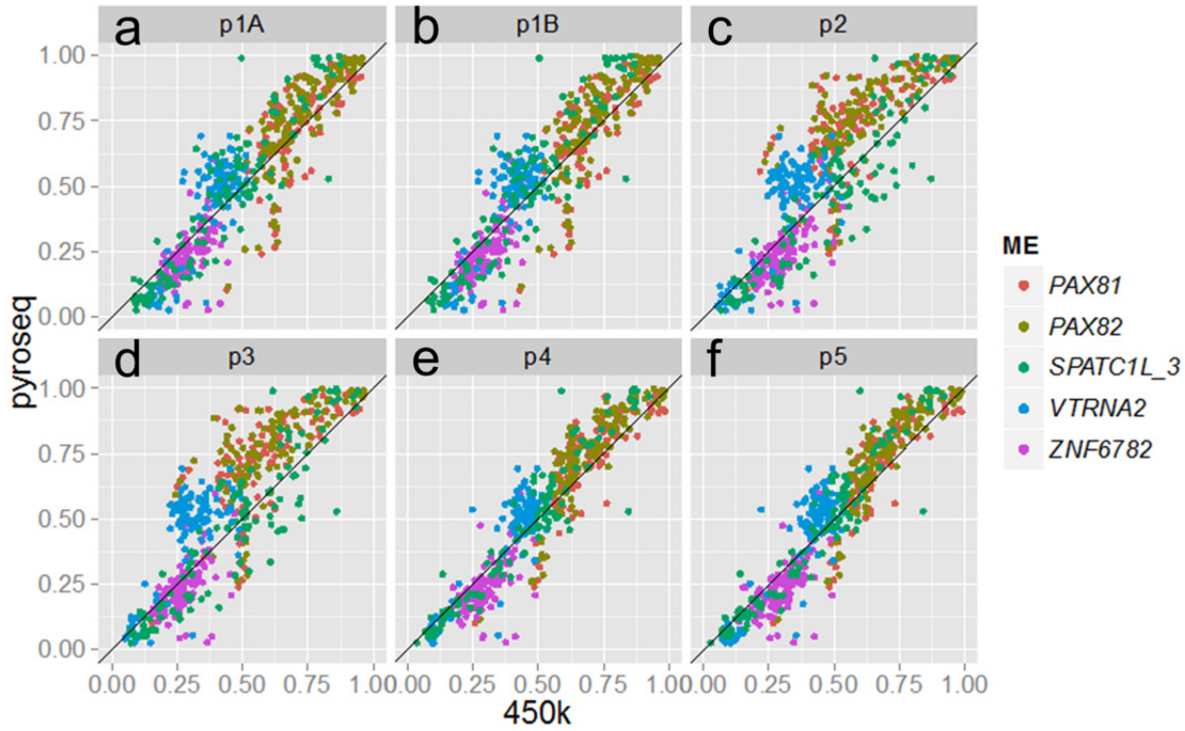

Supplementary Figure 3: Validation of 450k beta values and optimization of analysis pipeline. Comparison of % methylation estimates by pyrosequencing vs. 450k array beta values at five ME loci, for six different QC pipelines (see Supplementary Methods for details). Correlations are generated from 554 data points for each method, corresponding to loci measured in multiple individuals for which comparable 450k and pyrosequencing data are available. (**a**) Pipeline 1A, Pearson R (P)=0.89; Spearman R (S)=0.91. (**b**) Pipeline 1B, P=0.89; S=0.91. (**c**) Pipeline 2, P=0.86; S=0.84. (**d**) Pipeline 3, P=0.86; S=0.85. (**e**) Pipeline 4, P=0.92; S=0.94. (**f**) Pipeline 5, P=0.92; S=0.94.

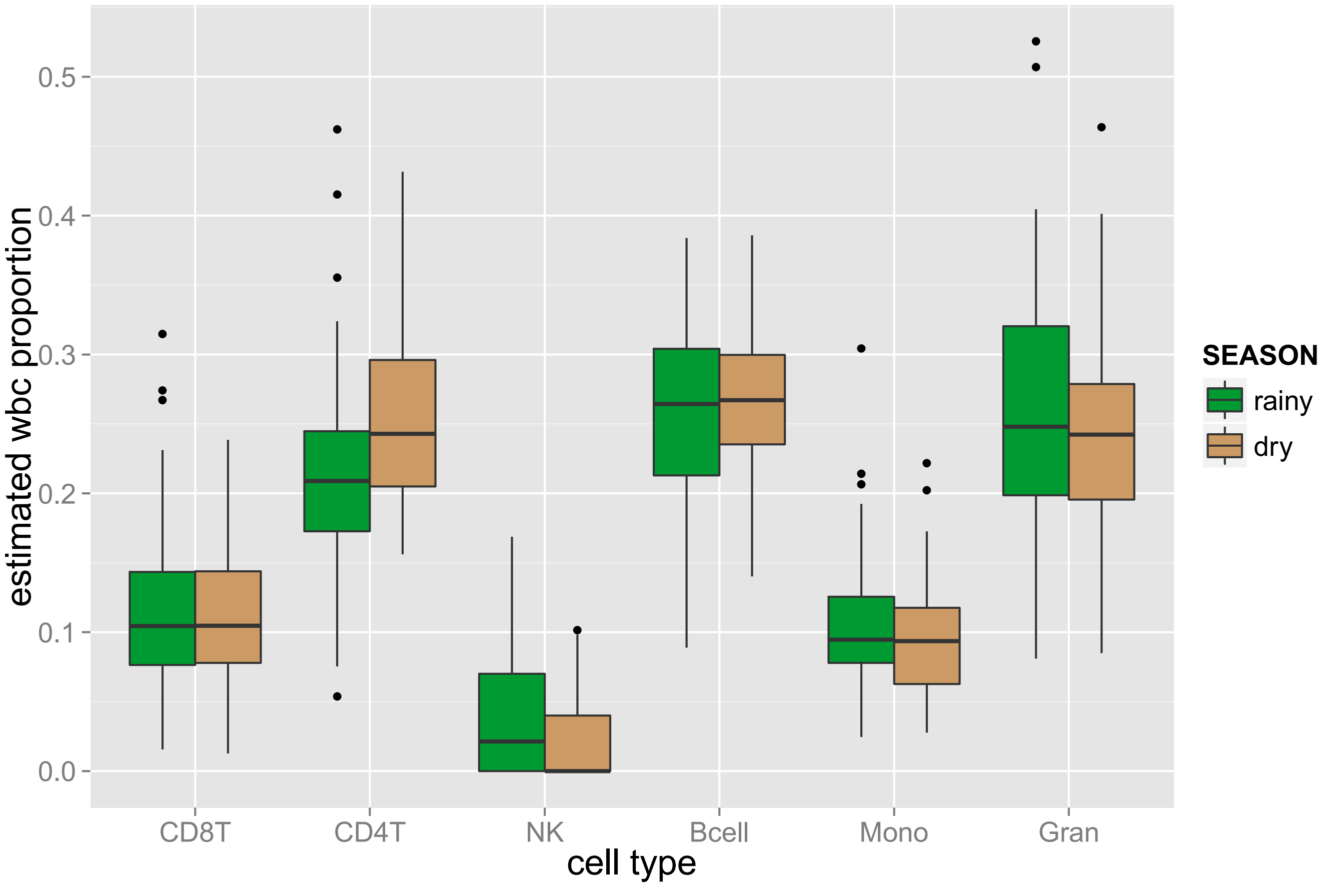

Supplementary Figure 4: Estimated white blood cell (WBC) fraction vs. WBC type. Estimates were derived from our 450k methylation data using the method of Jaffe & Irizarry (*Genome Biol* 2014). The results suggest subtle SoC effects on WBC composition, particularly for CD4T cells and NK cells. (CD8T: $CD8^+$ T cells, CD4T: $CD4^+$ T cells, NK: natural killer cells, Bcell: B cells, Mono: monocytes, Gran: granulocytes.)

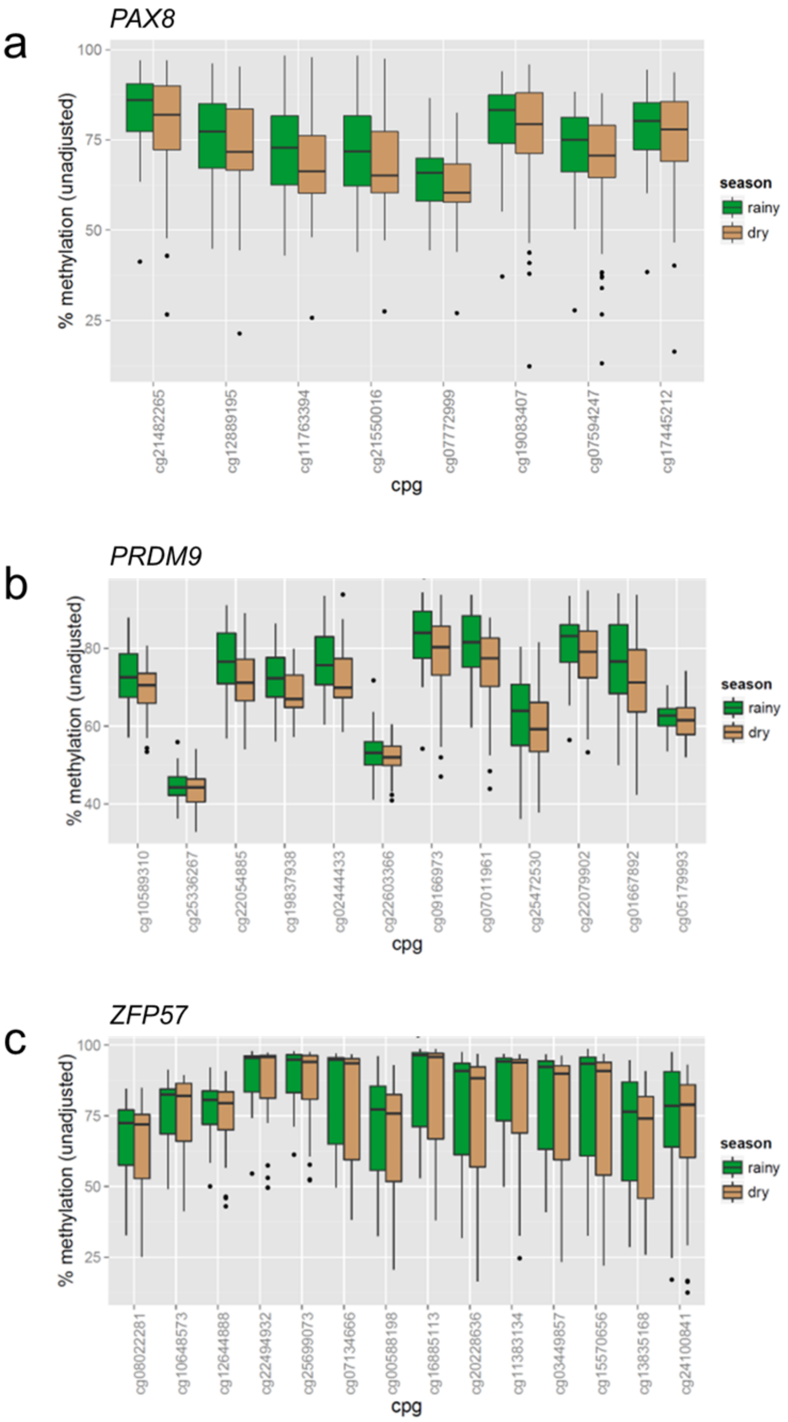

Supplementary Figure 5: Plots of methylation estimates (unadjusted beta values) of other top ranking DMRs associated with SoC in the 450k analysis. (**a**) *PAX8* (ranked 2nd). (**b**) *PRDM9* (ranked 3rd). (**c**) *ZFP57* (ranked 6th).

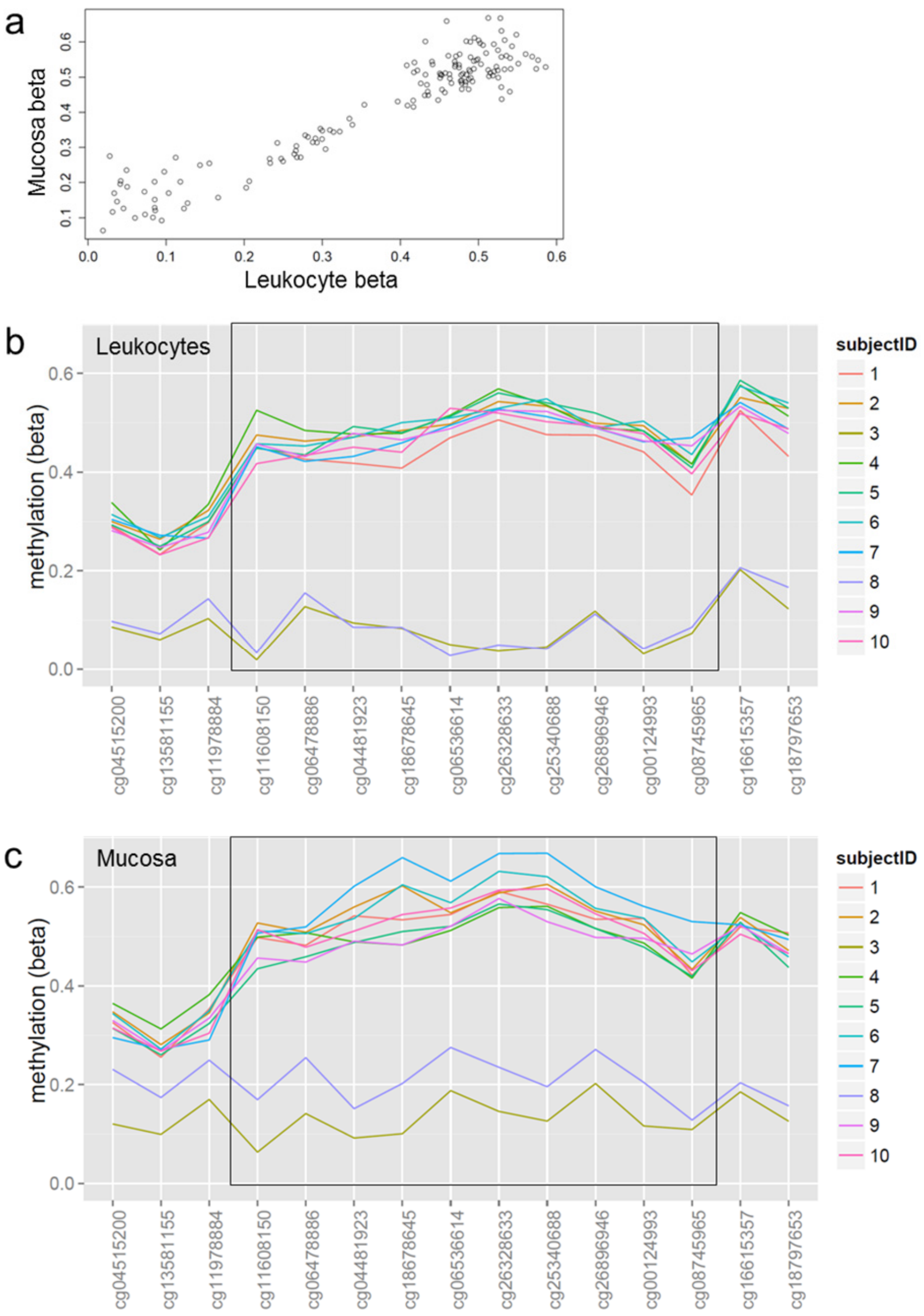

Supplementary Figure 6: Illumina 450k data on peripheral blood leukocytes and colonic mucosa (Harris et al., *Epigenetics* 2013) confirms systemic interindividual variation in DNA methylation across the entire *VTRNA2-1* imprinted DMR. (**a**) Among all 10 individuals studied, at the 15 probes in the *VTRNA2-1* SoC DMR, methylation in colonic mucosa is strongly correlated with that in peripheral blood leukocytes. (**b**) In leukocytes (GSE32148), two individuals (subjects 3 and 8) are hypomethylated across the entire *VTRNA2-1* imprinted DMR (box). (**c**) The same two individuals are hypomethylated in colonic mucosa (GSE32146).

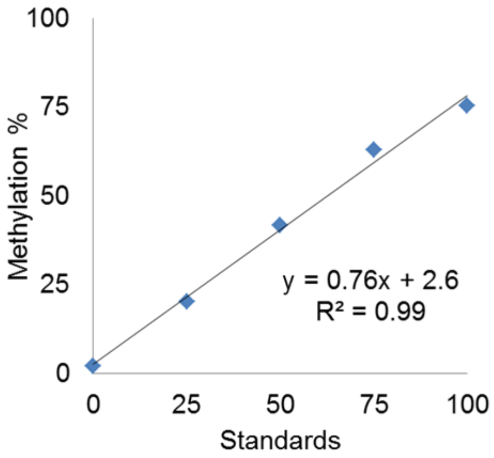

Supplementary Figure 7: Standard curve of the *VTRNA2-1* pyrosequencing assay.

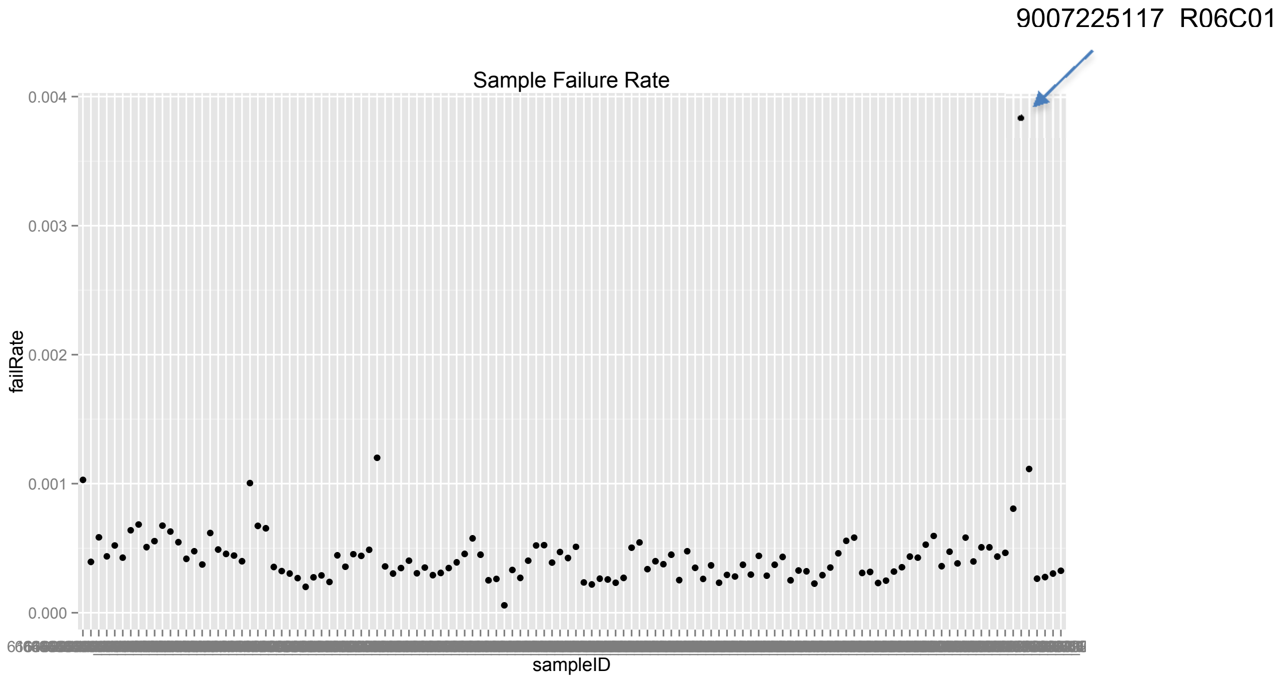

Supplementary Figure 8: Proportion of probes with detection p-value > 0.01 for each sample.

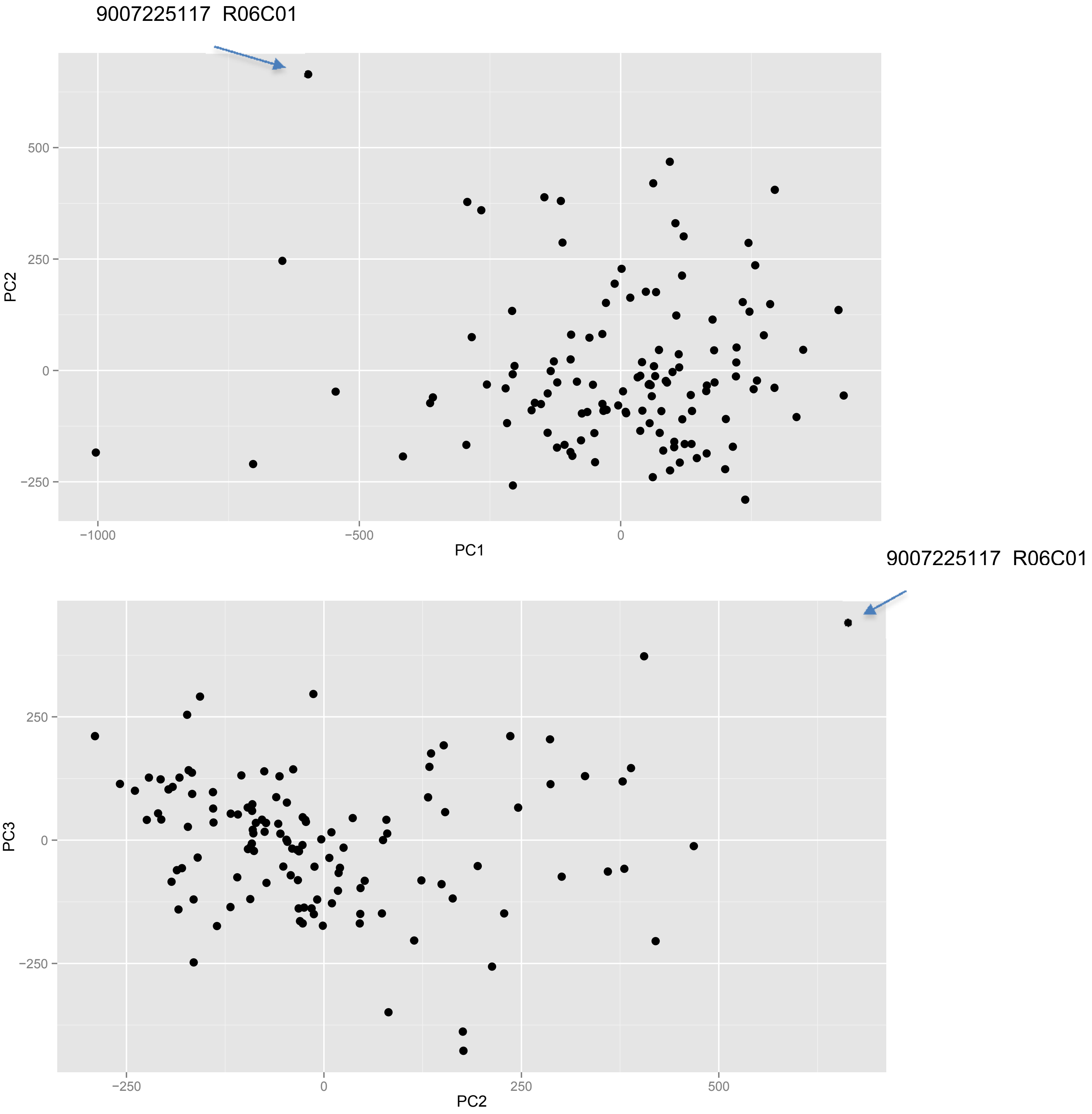

Supplementary Figure 9: PCA of array-wide methylation. Potential outlier is also sample with highest number of probes with detection p-value > 0.01 (see Supplementary Fig. 7). This probe was removed from subsequent analyses.

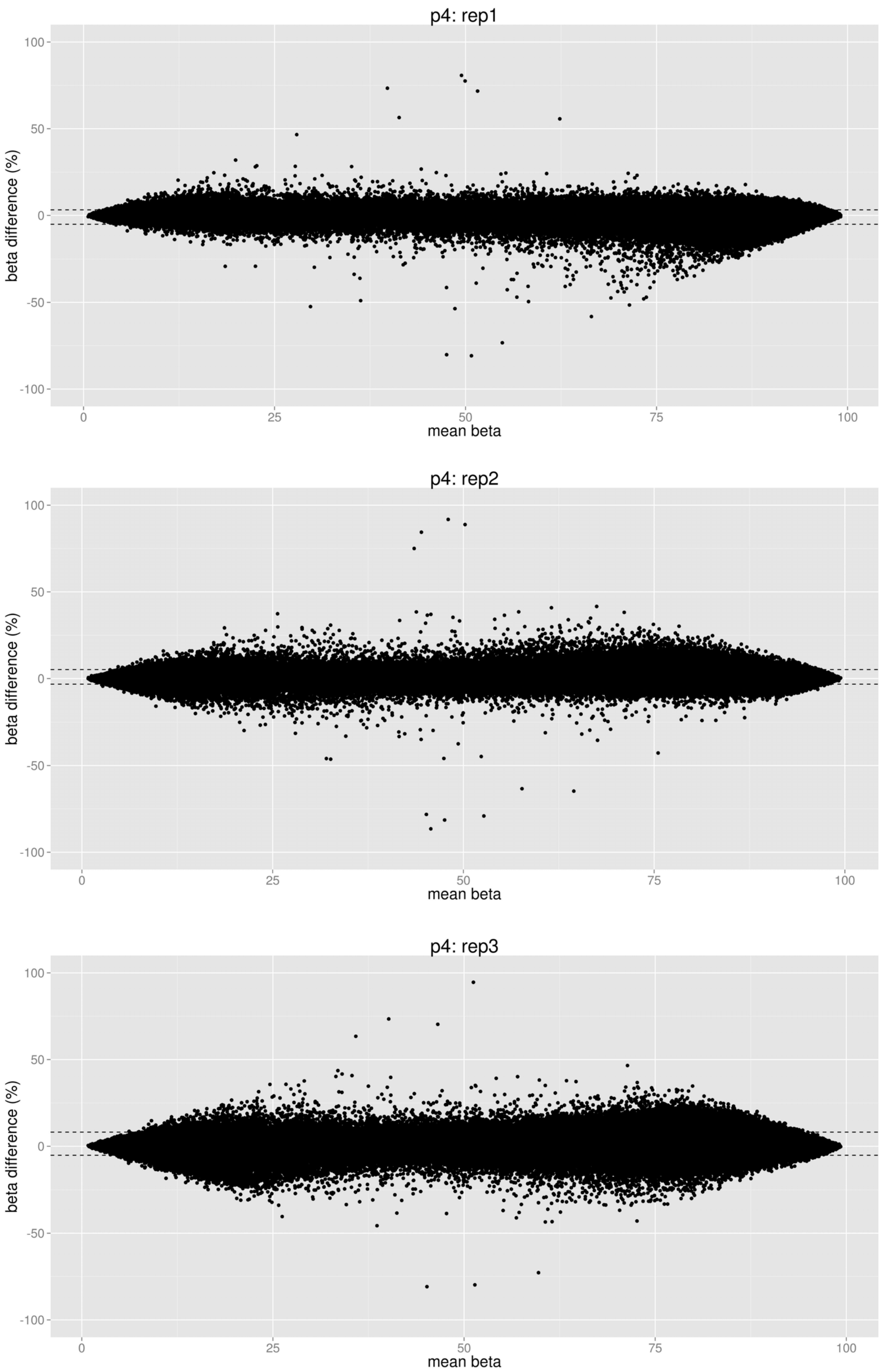

Supplementary Figure 10: Bland-Altman plots illustrate cross-replicate differences in methylation across the range of possible beta estimates, for each of the 3 replicates in the study. In these plots, probe-wise mean estimated beta values (across both replicates, x-axis) are plotted against differences in estimated betas (y-axis). 95% confidence intervals for the mean difference across all probes (mean +/- 2SD) are represented by the horizontal dotted lines.

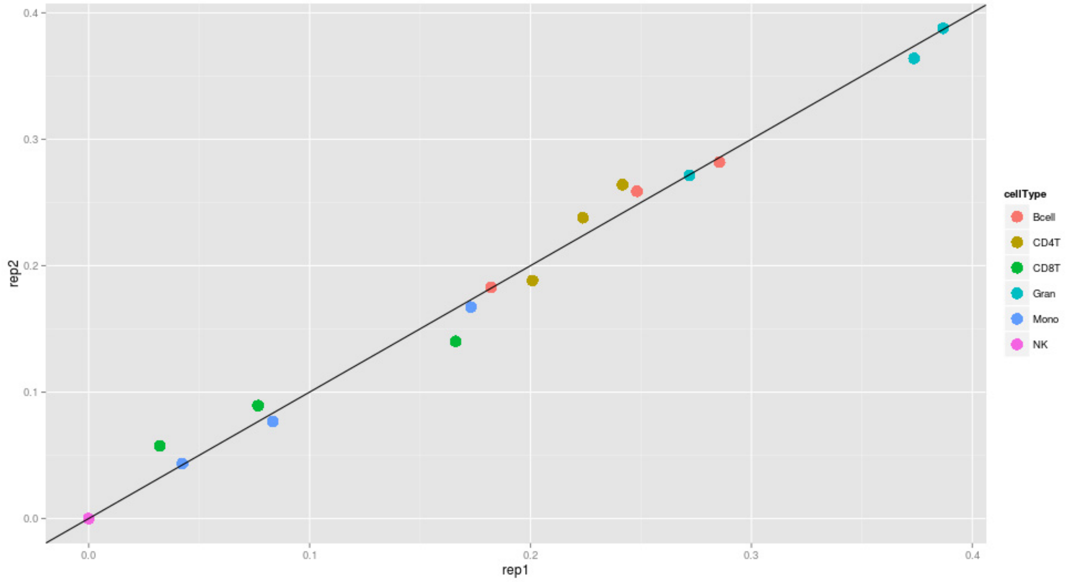

Supplementary Figure 11: Comparison of WBC estimates across two technical replicates. Spearman R=0.99, $p<10^{-8}$.
